# Physics-Informed Multi-Fidelity Graph Learning for Sequence-Aware Residual Bolt Preload Prediction in Hyperelastic-Sealed Flanges

**DOI:** 10.3390/s26175534

**Published:** 2026-08-31

**Authors:** Junyi Wang, Jiubin Tan, Yongmeng Liu, Jiacheng Zhong

**Affiliations:** 1 School of Instrumentation Science and Engineering, Harbin Institute of Technology, Harbin 150001, China; 2 Institute of AI for Industries, Chinese Academy of Sciences, Nanjing 211135, China; 3 Shenyang Institute of Automation, Chinese Academy of Sciences, Shenyang 110169, China

**Keywords:** graph neural network, residual bolt preload, tightening sequence, elastic interaction coefficient, multi-fidelity learning, aleatoric uncertainty, uncertainty quantification

## Abstract

**Highlights:**

**What are the main findings?**

- A physics-informed graph-learning workflow was developed for sequence-conditioned residual preload fields using EICM pre-training, nonlinear-discrepancy transfer, and ultrasonic measurement-based uncertainty quantification.
- On the augmented development split, TransformerConv-L3 achieved an RMSE of 170.9 N and 93.9% coverage for the nominal 95% prediction interval, outperforming the recurrent baselines evaluated under the same protocol.

**What are the implications of the main findings?**

- The full causal graph provides a compact computational representation of sequence-dependent bolt interactions and can be evaluated in milliseconds.
- The workflow integrates mechanics-informed simulation and ultrasonic measurements for sequence-aware preload prediction and uncertainty quantification.

**Abstract:**

Sequential tightening redistributes load among bolts and produces a residual preload field that depends on the complete operation history. This study presents a physics-informed graph-learning framework for a 60-bolt circular flange. The model receives initial preloads and tightening order and predicts the residual preload field on a complete causal directed graph. All 1770 admissible later-to-earlier edges are retained. Among them, 240 local edges carry non-zero Elastic Interaction Coefficient Method (EICM) attributes, while 1530 distant edges remain available to learned message passing. The multi-fidelity dataset combines online EICM responses, prescribed nonlinear-discrepancy labels, and repeated ultrasonic preload measurements. On the augmented development split, TransformerConv-L3 achieved an MAE of 138.3 N, an RMSE of 170.9 N, an NLL of 8.839, and 93.9% coverage for the nominal 95% prediction interval. Its RMSE was 56.9% lower than that of the strongest recurrent baseline evaluated under the same protocol. These results demonstrate the value of combining causal topology with mechanics-informed edge attributes for residual preload prediction.

## 1. Introduction

The sealing performance of a bolted flange depends on the residual clamping force established during assembly. Sequential tightening redistributes the load among bolts, so the final preload can differ from the applied initial preload. An uneven or insufficient residual distribution may reduce interface contact pressure and contribute to leakage or local fatigue loading [1,2,3]. Predicting the post-assembly preload distribution is, therefore, relevant to tightening-sequence design and assembly quality control.

Hyperelastic sealing rings add material and geometric nonlinearities, large deformation, and evolving contact states [4,5]. Elastic interactions among neighbouring bolts also make the final preload distribution dependent on tightening order [1,6,7]. Residual preload is, therefore, treated here as a coupled distribution across the flange rather than as independent bolt responses.

In practical measurements, residual preload is commonly inferred from ultrasonic measurements of bolt elongation. More generally, ultrasonic measurement accuracy depends on stable acoustic coupling at the probe–surface interface and on reliable interpretation of time-domain reflections, both of which can introduce observation variability when acquisition conditions change [8,9]. When such variability is ignored in bolt-preload inference, the resulting estimates can become overconfident, potentially concealing preload loss or prompting unnecessary maintenance. Quantifying measurement uncertainty is, therefore, important when ultrasonic observations are used to assess the condition of bolted joints.

Most sequence-aware preload models assume linear elastic behaviour. Studies of sealed flanges have examined nonlinear contact and material effects, but repeated nonlinear FE analyses remain costly when many tightening sequences must be evaluated. An efficient surrogate that represents bolt interactions and transfers information from elastic calculations to nonlinear simulations could, therefore, support assembly planning for hyperelastic-sealed flanges.

This study formulates the task as supervised prediction of the residual preload state after a prescribed tightening sequence. The inputs are the initial bolt preloads and tightening order, and the outputs are the final residual bolt preloads. Repeated ultrasonic preload measurements provide the targets for the heteroscedastic UQ head.

The framework combines a complete causal bolt graph, signed local EICM edge attributes, and progressive training across elastic, nonlinear-transfer, and ultrasonic-measurement stages within one residual-field surrogate.

The main contributions of this study are as follows:

- Sequence-aware residual preload surrogate: We formulate the coupled residual preload field of a 60-bolt flange as a graph regression problem conditioned on initial preload and tightening order.
- Physics-informed graph representation: We retain the full causal directed topology and use signed EICM coefficients as local edge attributes, allowing the network to condition message weights on both sequence direction and an interpretable linearized interaction prior.
- Progressive multi-fidelity training: We pre-train on EICM responses, transfer to prescribed nonlinear-discrepancy labels, and fit a heteroscedastic head to repeated ultrasonic preload measurements. The evaluation covers the 60-bolt configuration and the three tightening families.

## 2. Literature Review

### 2.1. Bolt Tightening and the Impact of Tightening Sequence on Preload

The reliability of bolted connections depends on preload control. Recent studies have addressed preload scatter using data-driven and control methods for individual bolts [2,3,10]. Multi-bolt flange joints also exhibit spatial coupling during tightening. Each operation deforms the joint and changes the residual preload of bolts tightened earlier, making the final distribution sequence dependent [1,6,7].

To predict and mitigate sequence-dependent preload loss, researchers have proposed analytical and numerical models based on linear superposition. The Elastic Interaction Coefficient Method (EICM), introduced by Bibel and Ezell [11], represents the multi-bolt flange as a network of linear springs. An elastic interaction matrix quantifies how tightening one bolt changes the preload of bolts tightened earlier. Under proportional and additive deformation, the matrix can be used to calculate initial tightening forces for a target residual preload. Fukuoka and Takaki [12] used three-dimensional FE simulations to extract interaction coefficients for pipe flanges. Yang et al. [13] subsequently used a Markov-theory-based model to optimize initial preloads. These methods remain dependent on the linear elastic assumption and are less suitable when compliant seals introduce pronounced nonlinear behaviour.

Hyperelastic sealing rings can introduce material and geometric nonlinearities, large deformation, and evolving contact states during sequential assembly [4,5]. These effects limit the accuracy of direct linear superposition. Nonlinear three-dimensional FE analysis can represent them, but evaluating each candidate tightening sequence separately is computationally expensive [5]. This motivates sequence-aware surrogates for hyperelastic-sealed joints.

### 2.2. Data-Driven Surrogate Models and Graph Neural Networks

Data-driven surrogate models offer one way to reduce repeated nonlinear FE evaluations. By learning input–output mappings from simulation data, such models can approximate structural responses at lower evaluation cost. For bolted joints, artificial neural networks and convolutional neural networks have been used to estimate preload states, examine loosening, and optimize assembly parameters [10]. Multiscale convolutional and recurrent networks have also been studied for damage identification across different structural components [14].

Standard neural networks often use grids or fixed-length vectors and do not explicitly represent the connectivity of a multi-bolt flange [15,16]. GNNs instead represent interacting components as nodes and edges and have been used as surrogates for physical systems [17,18,19,20]. Mesh-based GNNs have also been applied to hyperelastic materials and deformable solids [21,22].

Applying GNNs to sequence-aware prediction of hyperelastic-sealed joints is constrained by the availability of high-fidelity data [23,24]. Generating nonlinear FE results for a large number of tightening sequences is expensive, whereas a small single-fidelity dataset may not cover the relevant input space [25,26]. Multi-fidelity strategies provide a practical mechanism for combining abundant low-cost calculations with fewer high-cost nonlinear evaluations [27,28,29].

### 2.3. Uncertainty Quantification and Discrepancy Modelling in Industrial Measurement

Measurement uncertainty is not unique to ultrasonic sensing: model-based metrology studies show that data-analysis choices and uncertainty-propagation procedures can materially influence the reported uncertainty of indirectly inferred quantities [30]. Accordingly, deterministic bolt-preload outputs alone do not describe the variability in repeated ultrasonic observations. UQ methods can instead associate predictions with intervals or distributions, although the interpretation depends on which uncertainty sources are represented [31,32,33].

Multi-fidelity discrepancy modelling uses lower-cost simulations to establish a baseline and higher-fidelity data to correct the model in the region represented by those data [27,28].

Heteroscedastic regression is one approach to modelling data-dependent aleatoric variability [34,35]. It can estimate a conditional mean and variance from measurement-derived targets [36,37]. In this study, the heteroscedastic head models aleatoric variability in the ultrasonic measurements.

Related flange studies have also quantified clamp-load loss from elastic interaction and gasket creep [38] and developed metamodels for tightening-sequence design [39].

The literature leaves a specific gap between data-driven sensing and mechanically structured residual-field prediction. Xu et al. [10] infer single-bolt preload from hydraulic pressure and nut-angle signals, whereas recent multi-branch Transformer fusion, exemplified by MCFormer for bearing fault diagnosis [40], combines heterogeneous sensor representations for classification. Together, these studies establish valuable foundations for signal-based inference and feature fusion. The remaining need is continuous, sequence-conditioned regression of a coupled multi-bolt residual preload field that embeds elastic transfer as a graph prior and connects successive computational fidelity stages.

## 3. Materials and Methods

### 3.1. Multi-Fidelity Dataset Construction Strategy

The workflow contains three stages. Stage 1 generates EICM elastic responses online. Stage 2 applies a prescribed distance- and step-dependent discrepancy to the EICM result to form nonlinear-transfer labels. Stage 3 uses repeated ultrasonic preload measurements for uncertainty quantification. The construction and training procedure for each stage are described below, while dataset composition and evaluation results are reported in Section 4.

#### 3.1.1. Elastic Residual Preload Generator

**Figure 1:**
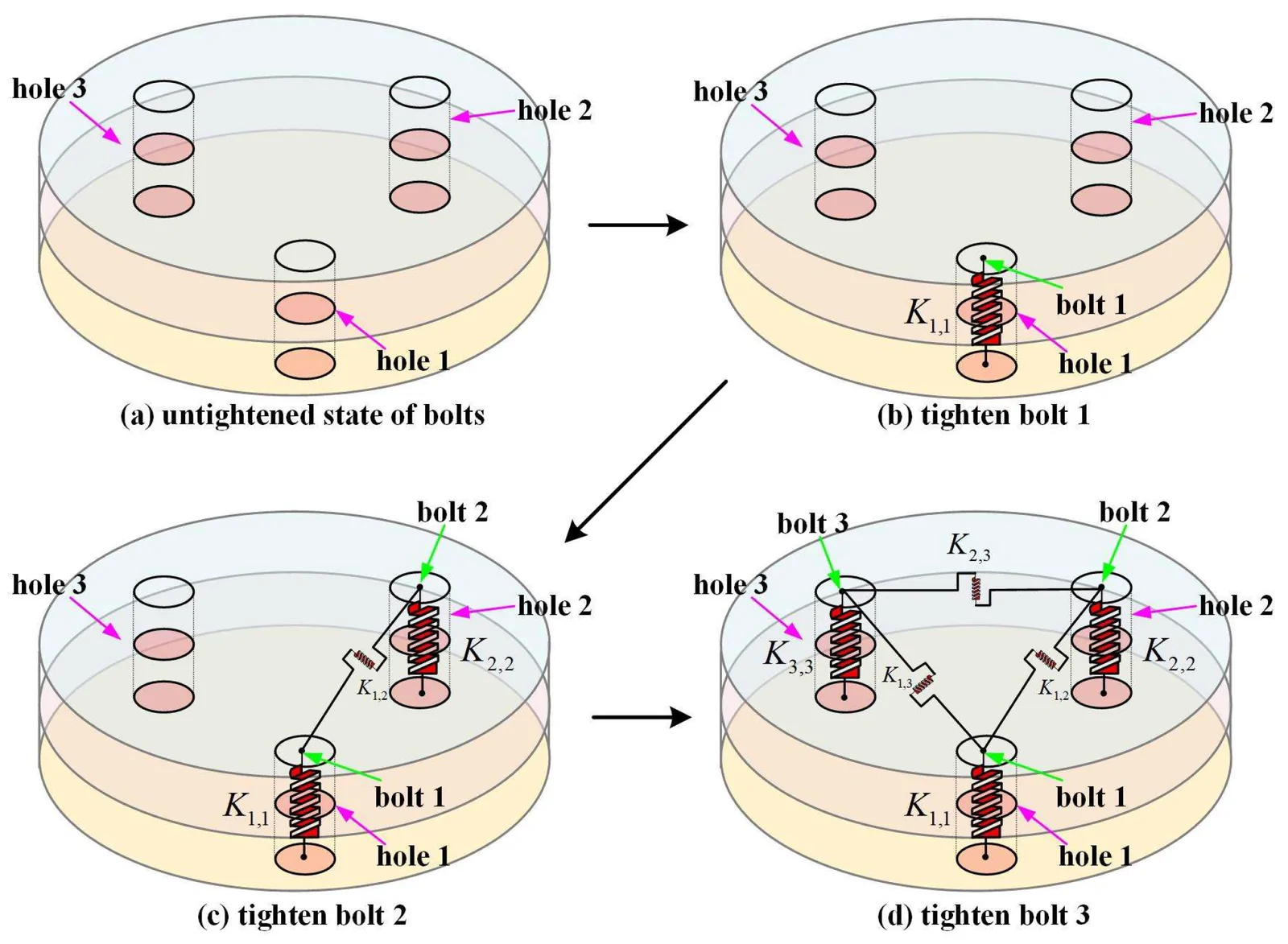
Elastic spring-node representation of the multi-bolt flange. Bolt indices, interaction compliances/stiffnesses and operation arrows identify the direction of sequence-dependent preload redistribution. Magenta arrows identify bolt holes, green arrows identify bolts, and black arrows indicate the tightening progression between states.

We developed an Elastic Residual Preload Generator to batch-generate preload data without sealing rings. This method is based on the EICM [7,11]. As shown in Figure 1, when all components are made of purely elastic materials, the multi-bolt flange system can be modelled as a spring-node system. In this system, the tightening of any single bolt induces elastic deformations that propagate through the flange structure to all other bolts, and this mechanical coupling can be fully described by a set of spring stiffness parameters.

Consider a flange system with n bolts tightened in a clockwise sequence 1→2→…→n. When the j-th bolt is tightened with a preload force $F_{j}$, the elastic deformation compatibility of all previously tightened bolts i=1,…,j−1 is governed by the following system of equilibrium equations:

(1) $$\left[\begin{array}{cccc} K_{1,1}^{-1}+K_{b_{1}}^{-1} & K_{1,2}^{-1} & \ldots & K_{1,j-1}^{-1}\\ K_{2,1}^{-1} & K_{2,2}^{-1}+K_{b_{2}}^{-1} & \ldots & K_{2,j-1}^{-1}\\ \vdots & \vdots & \ddots & \vdots \\ K_{j-1,1}^{-1} & K_{j-1,2}^{-1} & \ldots & K_{j-1,j-1}^{-1}+K_{b_{j-1}}^{-1} \end{array}\right]\left\{\begin{array}{c} \Delta S_{1,j}\\ \Delta S_{2,j}\\ \vdots \\ \Delta S_{j-1,j} \end{array}\right\}=-\left\{\begin{array}{c} K_{1,j}^{-1}F_{j}\\ K_{2,j}^{-1}F_{j}\\ \vdots \\ K_{j-1,j}^{-1}F_{j} \end{array}\right\}$$

where $\Delta S_{i,j}$ is the preload change in the *i*-th bolt induced by tightening of the *j*-th bolt; $K_{ij}^{-1}$ is the elastic compliance between bolt holes *i* and *j*; $K_{ii}^{-1}$ is the axial compliance of the *i*-th bolt hole, i.e., the clamped member compliance; and $K_{b_{i}}^{-1}$ is the axial compliance of bolt *i*.

Since all stiffness parameters are determined by the system structure, the compliance matrix above is constant for a given bolt configuration. Applying Cramer’s rule to solve for $\Delta S_{i,j}$ yields elastic deformation as we tighten sequentially from the first bolt to the *n*-th bolt:

(2) $$\Delta S_{i,j}=-\frac{|\begin{array}{ccccc} K_{1,1}^{-1}+K_{b_{1}}^{-1} & \ldots & K_{1,j}^{-1} & \ldots & K_{1,j-1}^{-1}\\ \vdots & \ddots & \vdots & & \vdots \\ K_{i,1}^{-1} & \ldots & K_{i,j}^{-1} & \ldots & K_{i,j-1}^{-1}\\ \vdots & & \vdots & \ddots & \vdots \\ K_{j-1,1}^{-1} & \ldots & K_{j-1,j}^{-1} & \ldots & K_{j-1,j-1}^{-1}+K_{b_{j-1}}^{-1} \end{array}|}{|\begin{array}{ccccc} K_{1,1}^{-1}+K_{b_{1}}^{-1} & \ldots & K_{1,i}^{-1} & \ldots & K_{1,j-1}^{-1}\\ \vdots & \ddots & \vdots & & \vdots \\ K_{i,1}^{-1} & \ldots & K_{i,i}^{-1}+K_{b_{i}}^{-1} & \ldots & K_{i,j-1}^{-1}\\ \vdots & & \vdots & \ddots & \vdots \\ K_{j-1,1}^{-1} & \ldots & K_{j-1,i}^{-1} & \ldots & K_{j-1,j-1}^{-1}+K_{b_{j-1}}^{-1} \end{array}|}\cdot F_{j}$$

where $\Delta S_{i,n}$ is the value of the change in preload force of the *i*-th bolt after preloading the *n*-th bolt; $S_{n}$ is the preload force applied to the *n*-th bolt; $S_{i,j}$ is the elastic interaction stiffness between the *i*-th and *j*-th bolts; $S_{i,i}$ is the axial stiffness of the *i*-th bolt hole; and $S_{bi}$ is the *i*-th bolt stiffness. The i-th column of the numerator determinant is replaced by the right-hand-side vector. Because both determinants depend only on the system geometry and material properties, their ratio is a constant for a fixed bolt configuration. Therefore, $\Delta S_{i,j}$ and $F_{j}$ are linearly related, which defines the elastic interaction coefficient $A_{i,j}$:

(3) $$\Delta S_{i,j}=A_{i,j}\cdot F_{j}$$

where the simplified closed-form expression for $A_{i,j}$ follows from the observation that off-diagonal coupling stiffnesses $K_{ij}$ (i≠j) are much smaller than the diagonal terms, so the determinant ratio reduces to the ratio of a single cross-stiffness to the sum of the diagonal and bolt axial stiffnesses.

For a system of n bolts, the cumulative effect of all subsequent tightening operations on each bolt can be superimposed under the linear elastic assumption. The relationship between the initial preload vector $S_{i}=[S_{i,1},S_{i,2},\ldots ,S_{i,n}{]}^{T}$ and the final residual preload vector $S_{f}=[S_{f,1},S_{f,2},\ldots ,S_{f,n}{]}^{T}$ is then expressed in compact matrix form:

(4) $$S_{f}=A\cdot S_{i}$$

where A is the n×n elastic coefficient matrix. Under a fixed tightening sequence, A is an upper triangular matrix with ones on the diagonal and elastic interaction coefficients $A_{i,j}$ (j>i) in the upper off-diagonal entries:

(5) $$A=\left[\begin{array}{ccccc} 1 & A_{1,2} & A_{1,3} & \ldots & A_{1,n}\\ 0 & 1 & A_{2,3} & \ldots & A_{2,n}\\ \vdots & & \ddots & \ddots & \vdots \\ 0 & \ldots & 0 & 1 & A_{n-1,n}\\ 0 & \ldots & \ldots & 0 & 1 \end{array}\right]$$

The upper triangular structure reflects the causal nature of the sequential tightening: only bolts tightened after bolt i can alter its residual preload.

The implemented topology is a complete causal directed graph. For a complete 60-bolt sequence, every admissible edge from a later-tightened bolt to an earlier-tightened bolt is retained, giving 1770 directed edges. EICM supplies non-zero coefficients to the four nearest positions on each circumferential side. This produces 240 edges (13.56%) with non-zero physical-prior attributes and 1530 distant edges with zero-valued physical attributes. All edges pass through the learned edge embedding and message-passing operator. Thus, locality applies to the mechanics-derived attribute rather than to graph connectivity. A matched mechanics analysis across several neighbourhood radii will quantify the associated long-range preload contribution.

For a specific tightening sequence $P=[p_{1},\ldots ,p_{n}]$, where $p_{k}$ denotes the physical bolt number tightened at the k-th step, the coefficient matrix $A^{\prime }$ must be re-indexed to reflect the actual tightening order. This process is achieved via a permutation-based basis transformation:

(6) $$A^{\mathrm{seq}}=E\cdot A^{\prime }\cdot E^{T}$$

where E is the permutation matrix defined by P. The final elastic coefficient matrix A is obtained by extracting the upper triangular part, i.e., $A=\mathrm{triu}(A^{\mathrm{seq}})$, preserving the causal constraint. Once A is determined, the initial preload required to achieve a desired uniform target residual preload $S_{f}^{*}$ can be directly computed by matrix inversion:

(7) $$S_{i}=A^{-1}\cdot S_{f}^{*}$$

Thus, the specific residual preload can be generated given the tightening sequence and initial preload, enabling efficient batch generation of elastic training data.

**Figure 2:**
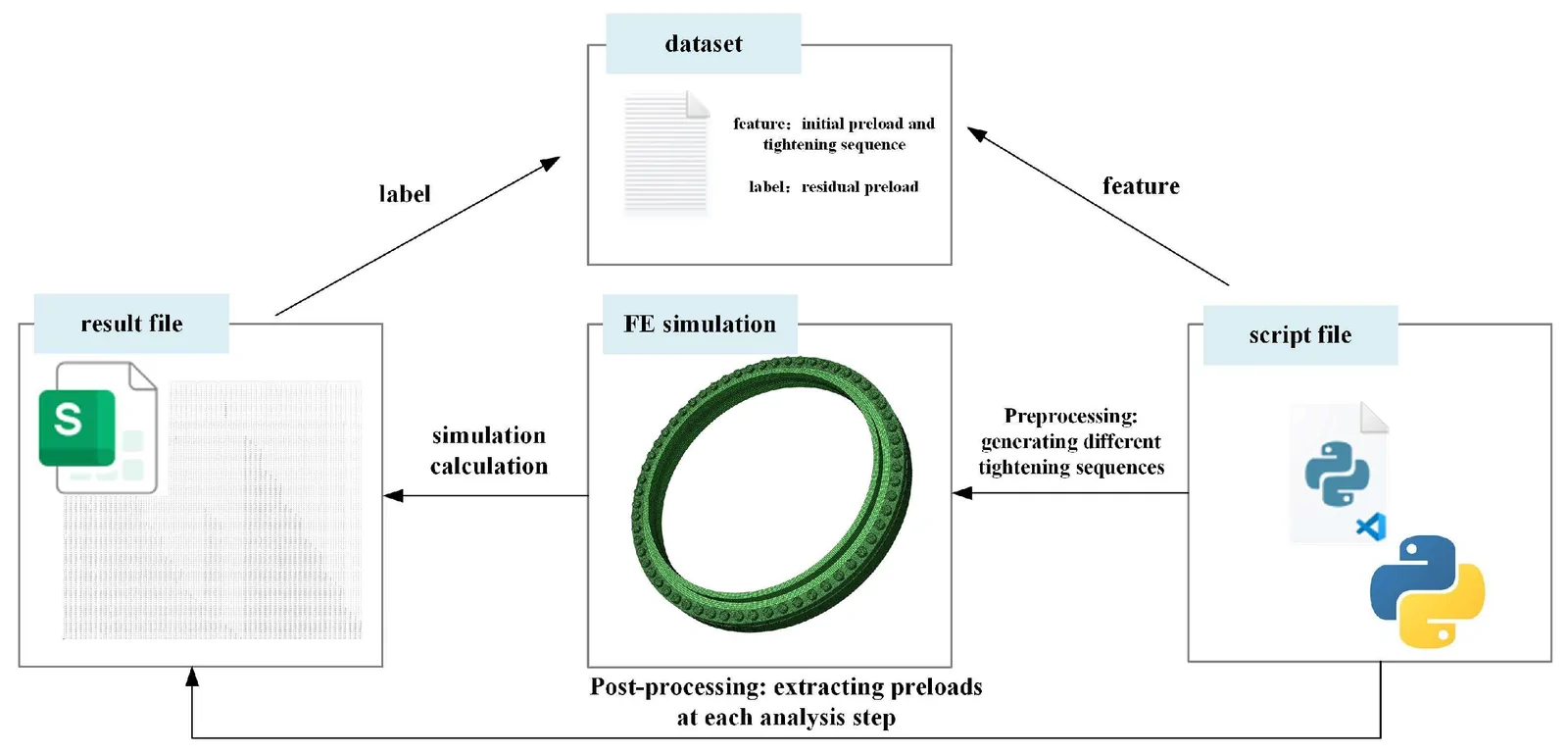
Data flow through the three-stage graph-learning workflow. Arrows indicate the transfer of model parameters and representations from elastic pre-training to nonlinear transfer and uncertainty quantification; dataset composition and split information are reported in Section 4.1.

The elastic residual preload generator batch-generates training tuples $\left(X,G,Y_{\mathrm{true}}\right)$ via the method described above. The training label $Y_{\mathrm{true}}$ is the true residual preload vector, the node feature X is the initial preload vector, and the graph structure G encodes the tightening sequence, as detailed in Section 3.2.1. Figure 2 summarizes the data construction workflow.

#### 3.1.2. Prescribed Nonlinear-Discrepancy Stage and Motivating FE Specification

**Figure 3:**
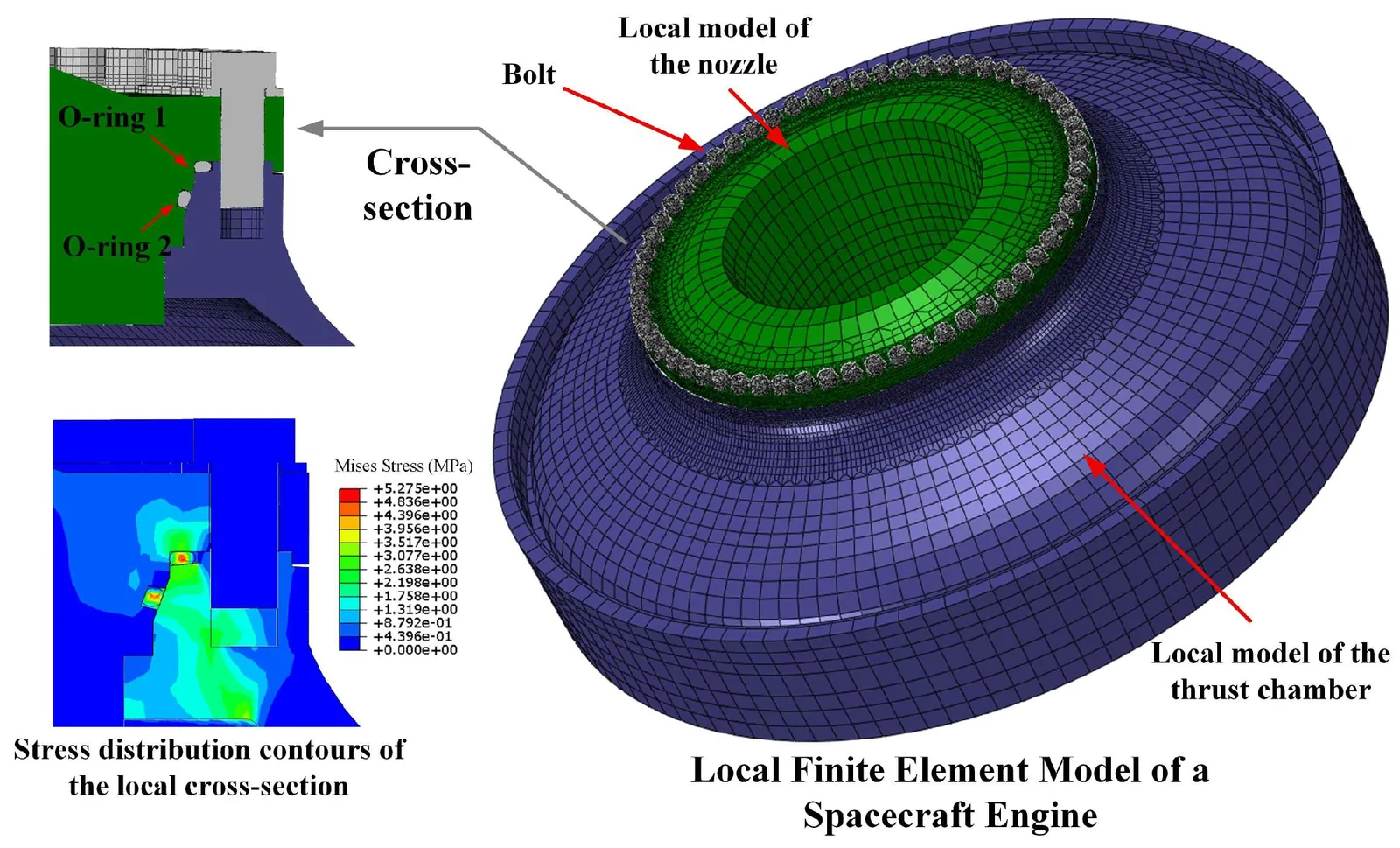
Sealed-joint geometry and representative stress visualization used to illustrate the motivating finite-element configuration.

The 60-bolt sealed pressure-joint geometry shown in Figure 3 defines the motivating application. The nonlinear-transfer stage introduces a prescribed discrepancy relative to the elastic EICM baseline so that the learning architecture can represent non-elastic corrections within the computational workflow.

The predicted response is the residual axial shank force after each bolt receives a prescribed preload; torque-to-preload conversion is not part of the input–output mapping. The motivating FE specification, therefore, omits explicit helical threads and represents the fastener through its global axial load path. This choice is consistent with Wu et al. [41], who directly prescribed bolt preload in a thread-free flange model and reported negligible friction effects on axial deformation in the tensile cases examined. Detailed thread geometry is still required when torque-angle response, local flank contact, thread-root stress, or self-loosening is the quantity of interest.

The finite-element specification uses Abaqus/Standard 2023 with geometrically nonlinear quasi-static analysis, C3D8R elements for metallic parts, and C3D8H hybrid elements for incompressible seals. A nominal discretization is used to define the motivating model.

The contact model uses finite-sliding surface-to-surface hard contact and an isotropic penalty friction law with a nominal coefficient μ = 0.10. For the force-prescribed axial problem considered here, this coefficient governs interface contact rather than torque-to-preload conversion. The selected value, therefore, defines the nominal axial redistribution benchmark, while friction-sensitive torsion, slip, loosening, and installation scatter lie outside the present response variable.

The finite-element workflow consists of an initial flange closure analysis followed by a sequential bolt-load analysis. The deformed seal contact state from the closure step is transferred to the tightening step so that the bolt sequence is applied to the assembled joint configuration.

Table 1 lists the nominal Mooney–Rivlin parameterization used to define the motivating seal specification.

**Figure 4:**
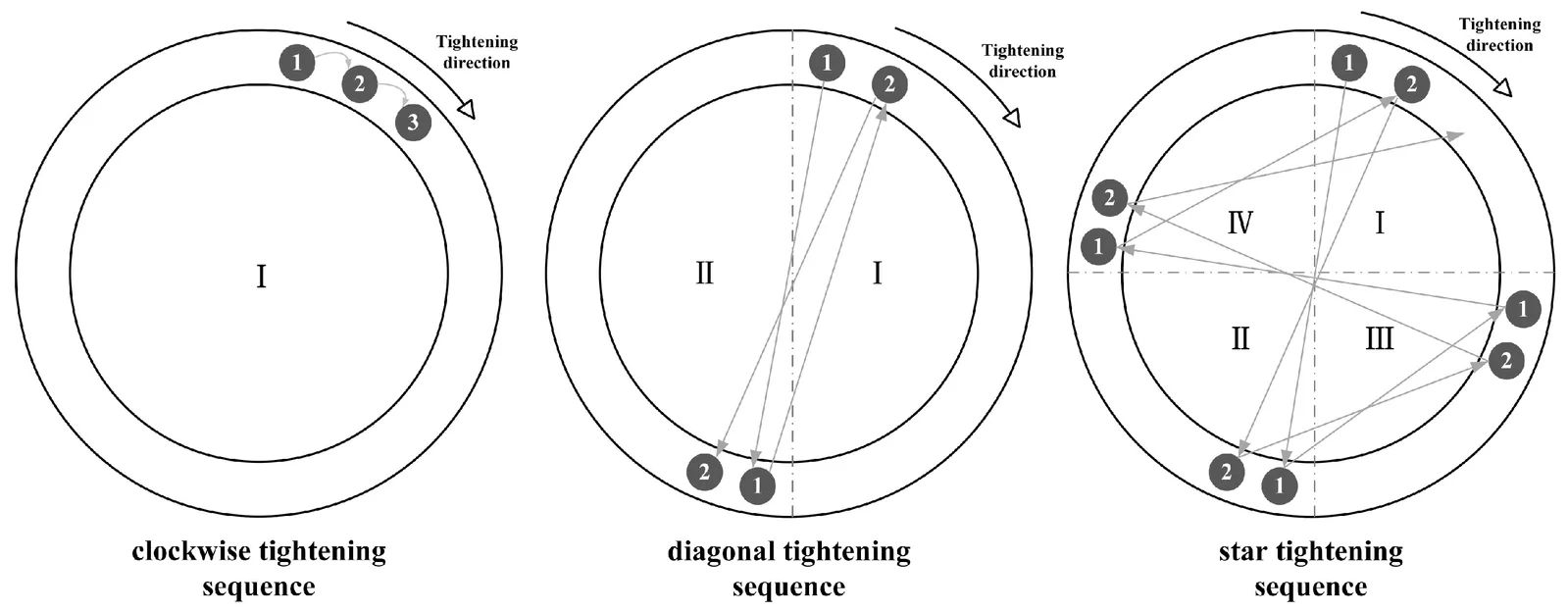
Schematic clockwise, diagonal and star tightening families. Directional arrows and representative bolt labels indicate tightening order; cyclic rotations supply augmented starting positions. Arabic numerals indicate representative tightening steps, whereas Roman numerals denote the partitioned tightening regions.

Three tightening sequence families define the sequence augmentation procedure illustrated in Figure 4:

- Clockwise tightening sequence: The entire flange is treated as a single region, and bolts are tightened consecutively in the circumferential direction.
- Diagonal tightening sequence: The flange is divided into two semi-circular regions. Bolts are tightened alternating diagonally between the two opposing regions.
- Star tightening sequence: The flange is partitioned into four quadrants. Bolts are tightened in a cross-diagonal star pattern across the four regions to distribute incremental seal compression.

For a nominally axisymmetric circular arrangement, cyclic rotation is applied to the clockwise, diagonal, and star sequence families as a data augmentation rule.

Let P represent a tightening-sequence vector and y the associated computational label. A cyclic shift operator Ck rotates the sequence forward by k positions:

(8) $$C^{k}(P)=[p_{(1+k)\%N},p_{(2+k)\%N},\ldots ,p_{(N+k)\%N}]$$

where % denotes the modulo operation with indices adjusted to standard 1-to-N numbering. Based on the structural symmetry, applying this physical shift to the tightening sequence results in an equivalent cyclic shift in the resulting residual preload vector:

(9) $$F(C^{k}(P))=C^{k}(F(P))$$

The generator creates cyclic and intermediate variants using this symmetry assumption. The corresponding dataset composition and partitioning are reported in Section 4.1.

#### 3.1.3. Uncertainty Quantification Stage Based on Ultrasonic Measurements

The UQ stage models input-dependent variability in ultrasonic preload measurements using a heteroscedastic output head. The predictor inputs remain the initial preload values and tightening order.

**Figure 5:**
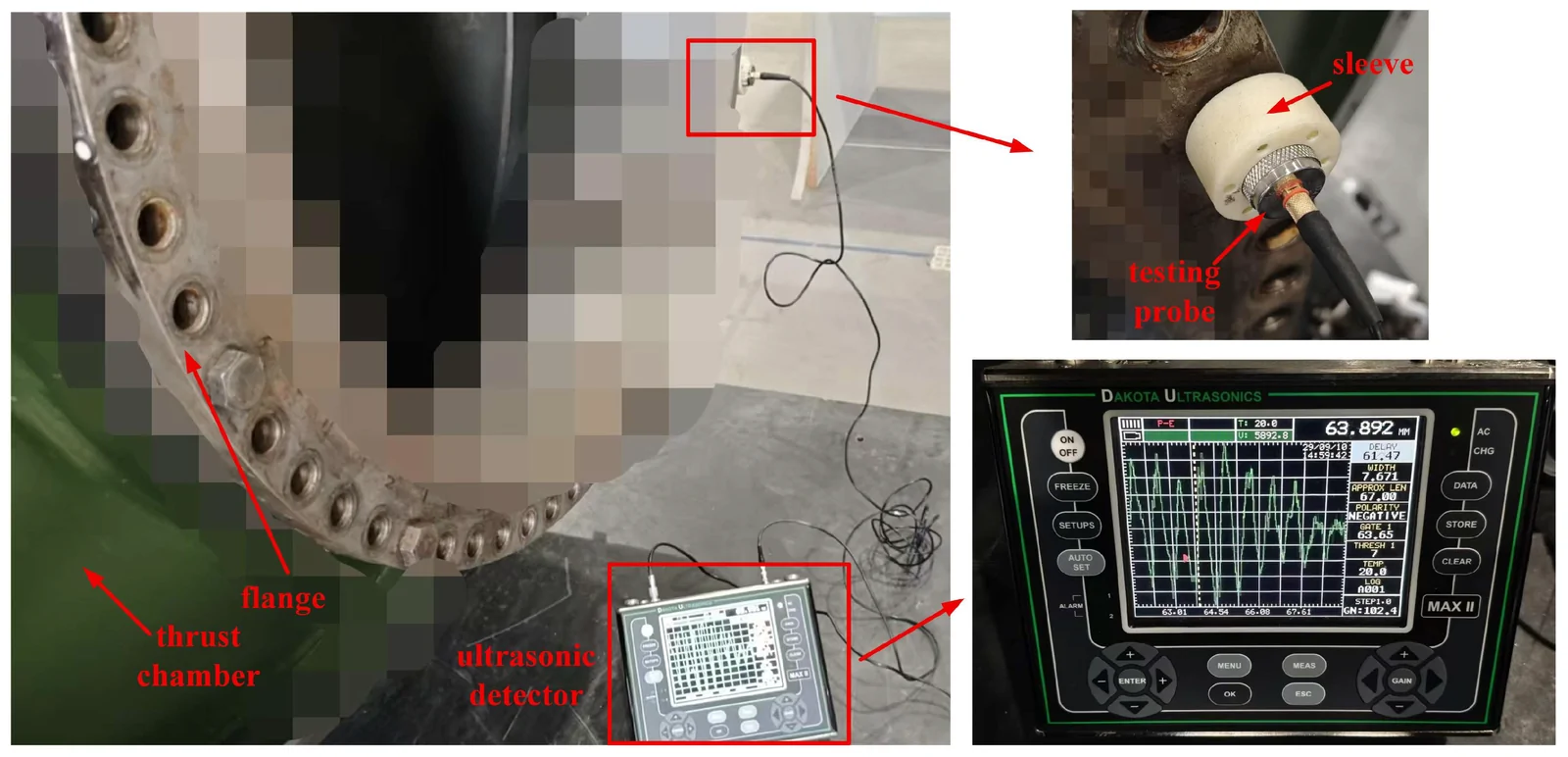
Ultrasonic preload measurement setup with a positioning sleeve, bolt-axis propagation path, and measurement direction.

Physical residual-preload data were obtained using a MAX II Ultrasonic Bolt Tension Monitor (Dakota Ultrasonics, Scotts Valley, CA, USA). As shown in Figure 5, a positioning sleeve controls probe placement and the ultrasonic propagation path follows the bolt axis.

Time-of-flight information was used to estimate bolt elongation and preload.

Five repeated ultrasonic measurements were collected for each bolt and condition and used as targets for UQ-head training. Dataset composition is reported in Section 4.1.

### 3.2. Construction of the GNN-Based UQ Network

**Figure 6:**
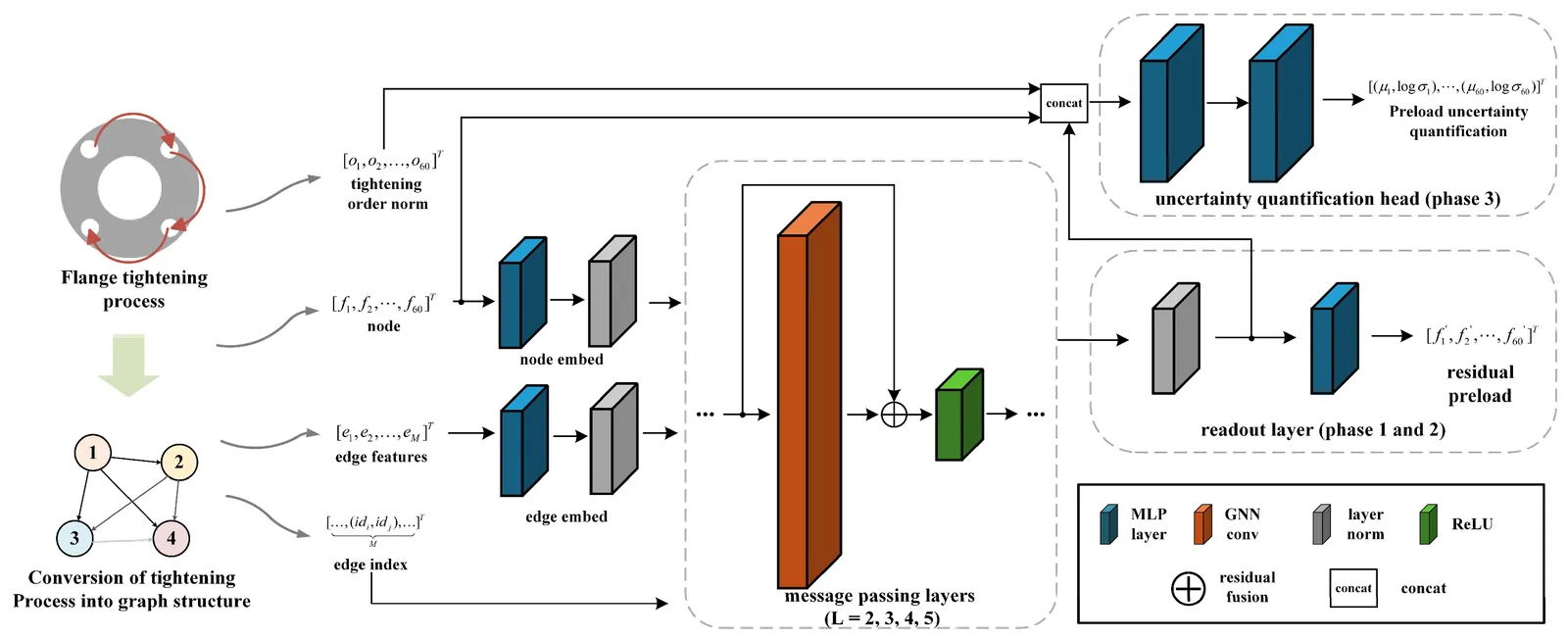
Sequence-aware graph network and three computational training phases. Colour-coded blocks distinguish elastic pre-training, nonlinear transfer, and UQ-head fitting; the backbone is frozen during the final phase. Red arrows indicate the tightening action, the green arrow indicates conversion from the physical process to the graph representation, numbers 1–4 denote example bolt/node indices, and block colors correspond to the layer types defined in the legend. Ellipses denote repeated intermediate message-passing blocks rather than missing content.

Figure 6 summarizes the graph-based prediction framework.

#### 3.2.1. Graph Representation of the Bolted Joint

The sequential tightening of a multi-bolt flange introduces complex spatial and temporal coupling. To capture this, the physical system is abstracted into a directed graph, where the topology reflects the sequential tightening operations. Each bolt in the flange is represented as a node. The input node feature vector, denoted as $[f_{1},f_{2},\ldots ,f_{N}{]}^{T}$, comprises the normalized initial applied preloads. The tightening sequence dictates the directional dependency between bolts. Specifically, a directed edge is established from node j to node i if bolt j is tightened after bolt i. This represents the physical phenomenon where the subsequent tightening of bolt j alters the residual preload of the already-tightened bolt i. This topological relationship is captured by the edge index matrix $[\ldots ,(id_{i},id_{j}),\ldots {]}^{T}$.

$[e_{1},e_{2},\ldots ,e_{M}{]}^{T}ji[o_{1},o_{2},\ldots ,o_{N}{]}^{T}$. The edge feature vector assigns an elastic interaction coefficient to each directed edge from bolt to bolt. These coefficients encode the linearized magnitude and direction of bolt-to-bolt coupling. The nonlinear seal contribution is introduced through the discrepancy-transfer stage, while the normalized tightening order supplies the sequence context.

#### 3.2.2. Unified GNN Backbone Architecture

The core predictive engine of our framework employs a unified “Embedding—Message Passing” architecture, designed to process the graph-structured input regardless of the specific downstream task.

1. Embedding Layer: Node and edge features are processed through separate embedding blocks. Each block consists of a multi-layer perceptron (MLP) followed by layer normalization.
2. Message-Passing Layers: The embedded features and graph topology are fed into a stack of L message-passing layers, where L∈{2,3,4}. Each layer performs neighbourhood aggregation using one of the five GNN convolution operators: TransformerConv, GATConv, GINConv, SAGEConv, or LinearConv. To mitigate the vanishing gradient problem and prevent over-smoothing, a residual connection fuses the block input with its output, followed by a ReLU activation before the next layer.

#### 3.2.3. Phase-Specific Output Branches

Our progressive multi-fidelity training strategy utilizes different output heads depending on the training phase, as depicted in the right section of the architecture diagram.

**Deterministic Readout Layer (Phases 1 and 2):** During the elastic pre-training (Phase 1) and nonlinear transfer learning (Phase 2), the network acts as a deterministic surrogate model. The output from the final message-passing layer is fed into a readout block, comprising layer normalization and an MLP. This branch maps the high-dimensional node representations directly to the predicted scalar residual preloads, $[f_{1}^{\prime },f_{2}^{\prime },\ldots ,f_{N}^{\prime }{]}^{T}$, which are optimized using standard regression loss functions against deterministic simulation data.

**Uncertainty Quantification Head (Phase 3):** In the final UQ phase, the GNN backbone is frozen to preserve the learned physical priors. A UQ head is activated. It receives a concatenation of three components: the frozen hidden representations from the GNN, the original normalized node features, and the normalized tightening order.

This concatenated tensor is processed by an MLP for probabilistic regression. For each bolt i, the UQ head outputs a conditional mean μi and log standard deviation log σi. The head is fitted to the ultrasonic measurement targets by minimizing the heteroscedastic NLL loss:

(10) $$L_{\mathrm{NLL}}=\frac{1}{N}\sum_{i=1}^{N}\left[log\sigma_{i}+\frac{(y_{i}-\mu_{i}{)}^{2}}{2\sigma_{i}^{2}}\right]$$

Here, *σ_i_* > 0 is the modelled observation scale about conditional mean μi. The resulting prediction intervals represent conditional aleatoric variability in the ultrasonic measurements.

### 3.3. Baseline Sequential Models: RNN and LSTM

For comparison, we use four sequential baseline models that treat multi-bolt tightening as a one-dimensional series. These recurrent models process bolts in tightening order and receive no explicit graph topology.

#### 3.3.1. Input Representation for Sequential Models

For the sequential baselines, the bolt tightening process is reformulated as a sequence of length N, where each time step corresponds to a bolt tightened in chronological order. At time step t, the input feature vector $x_{t}$ is constructed differently depending on the model variant.

For the Enhanced variants, the input incorporates physics-informed features:

(11) $$x_{t}={\left[\frac{f_{t}}{f_{max}},\;\frac{t}{N},\;\frac{A_{t,t-1}}{A_{max}}\right]}^{T}\in R^{3}$$

where $f_{t}$ is the initial preload of the bolt tightened at step t, t/N is the normalized tightening order, and $A_{t,t-1}$ is the elastic interaction coefficient between the current bolt and the previously tightened bolt. For the Base variants, only the first two features are retained:

(12) $$x_{t}={\left[\frac{f_{t}}{f_{max}},\;\frac{t}{N}\right]}^{T}\in R^{2}$$

#### 3.3.2. Elman RNN Architecture

The Elman recurrent neural network (RNN) updates the hidden state at each time step t as:

(13) $$h_{t}=tanh\;\left(W_{ih}\;x_{t}+W_{hh}\;h_{t-1}+b_{h}\right)$$

where $W_{ih}$ and $W_{hh}$ are the input-to-hidden and hidden-to-hidden weight matrices, and $b_{h}$ is a bias vector. RNN-Enhanced uses a bidirectional two-layer architecture, concatenating forward and backward states as $h_{t}=[{\vec{h}}_{t}∥{\overset{\leftarrow }{h}}_{t}]$; RNN-Base uses a single unidirectional layer.

#### 3.3.3. LSTM Architecture

The long short-term memory (LSTM) network extends the RNN with three gates to mitigate the vanishing gradient problem. Denoting $z_{t}=W_{x}\;x_{t}+W_{h}\;h_{t-1}+b$ as the pre-activation for the respective gate, the LSTM updates at step t are:

(14) $$\begin{array}{cc} i_{t},\;f_{t},\;o_{t}\; & =\sigma \;\left(z_{t}^{\left(i\right)},\;z_{t}^{\left(f\right)},\;z_{t}^{\left(o\right)}\right)\\ c_{t}\; & =f_{t}⊙c_{t-1}+i_{t}⊙\tanh\left(z_{t}^{\left(g\right)}\right)\\ h_{t}\; & =o_{t}⊙\tanh(c_{t}) \end{array}$$

where σ(⋅) is the sigmoid function, ⊙ the element-wise product, and $i_{t}$, $f_{t}$, $o_{t}$ are the input, forget, and output gates. LSTM-Enhanced uses a bidirectional two-layer architecture with hidden dimension $d_{h}=64$; LSTM-Base uses a single unidirectional layer.

#### 3.3.4. Output and Uncertainty Quantification

For both RNN and LSTM variants, the hidden state at each time step is mapped to a scalar predicted residual preload through a readout MLP:

(15) $${\hat{f}}_{t}=\mathrm{MLP}(h_{t})\in R$$

For the uncertainty quantification phase, the same frozen-backbone strategy is adopted. The pre-trained recurrent backbone is frozen, and a separate UQ head receives the frozen hidden state, original input features, and normalized tightening order. It predicts (μ_t, log σ_t), comprising a conditional mean and log standard deviation, for each bolt and is trained using the heteroscedastic NLL loss defined above. This design provides a consistent comparison with the proposed GNN framework across all three training phases.

## 4. Results and Discussion

### 4.1. Data Preparation and Experimental Setup

The implemented graph for a complete 60-bolt sequence contains all 1770 causal directed edges. The four nearest bolt positions on each circumferential side supply the eight non-zero EICM coefficient classes, producing 240 non-zero prior edges. The remaining 1530 edges retain zero-valued physical attributes and continue through the learned edge embedding. Table 2 lists the eight non-zero coefficient values, and the reported edge counts characterize the sparsity of the mechanics-informed prior.

Phase 1 generates 50 EICM samples per epoch for 150 epochs (7500 online samples). The nonlinear-transfer dataset contains 1620 augmented instances spanning three strategies, 60 starting positions, three intermediate lengths, and three preload variants. The UQ dataset contains 1620 augmented instances spanning three strategies, 60 starting positions, and nine preload variants, with five ultrasonic measurement replicates per instance. The nonlinear-transfer labels are produced by the prescribed discrepancy stage, and the UQ targets are the ultrasonic residual-preload measurements described in Section 3.1.3. Model development used a random 1458/162 augmented-sample partition; all three strategy families are represented in both subsets. The metrics reported below refer to this development split.

Model training was conducted on a workstation with an NVIDIA GeForce RTX 5070 GPU (NVIDIA Corporation, Santa Clara, CA, USA) using Adam. The settings in Table 3 produced the reported exploratory architecture comparison. The held-out development subset supported architecture and depth selection; the resulting values are, therefore, reported consistently as development metrics.

### 4.2. Comparison of GNN Architectures and Network Depths

**Figure 7:**
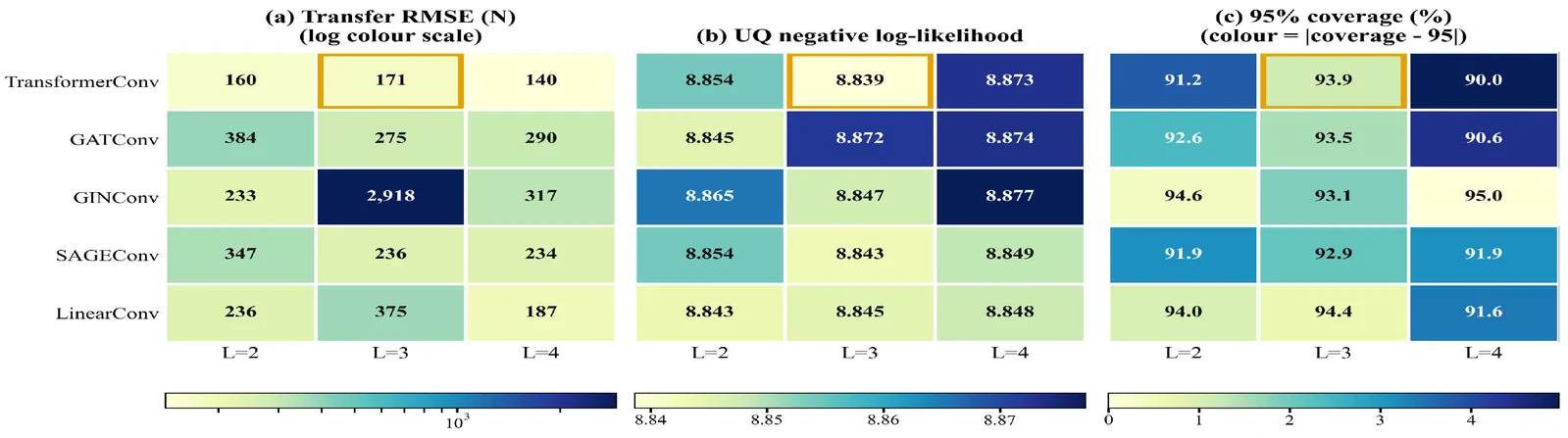
Development-split heatmaps of transfer RMSE, UQ NLL and nominal 95% coverage for one training run. Exact cell values are shown, and TransformerConv-L3 is identified as the selected balance across the three metrics. The gold outline marks the selected TransformerConv-L3 configuration in each panel.

**Figure 8:**
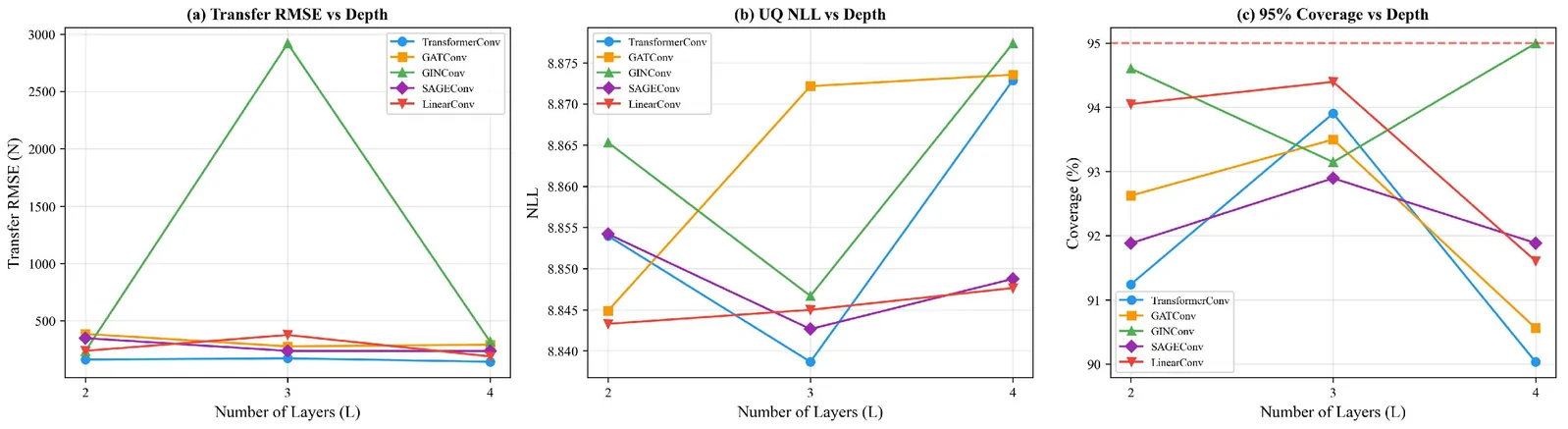
Single-run development-split metrics versus message-passing depth for five GNN operators. Distinct markers and line styles identify each architecture; values were used for exploratory model selection rather than untouched testing. The horizontal dashed line in the coverage panel denotes the nominal 95% coverage target.

To compare message-passing mechanisms and receptive-field depths, we evaluated five GNN architectures—TransformerConv, GATConv, GINConv, SAGEConv, and LinearConv—with two to four layers. Figure 7 summarizes the performance metrics, and Figure 8 shows their variation with network depth.

**Figure 9:**
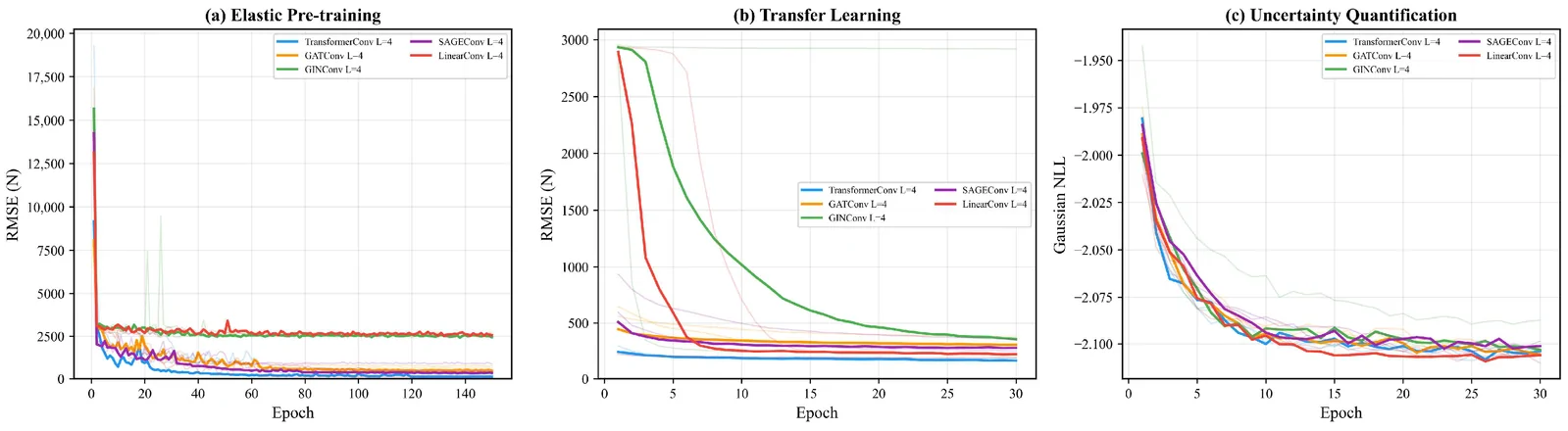
Training curves across EICM pre-training, nonlinear-discrepancy transfer, and UQ-head fitting with ultrasonic measurements. TransformerConv and GATConv stabilize after approximately 40–50 elastic epochs and SAGEConv after approximately 60 epochs under the prescribed schedule.

Figure 9 shows different elastic pre-training behaviour across architectures. TransformerConv and GATConv stabilized after approximately 40–50 epochs, while SAGEConv stabilized after approximately 60 epochs. GINConv and LinearConv plateaued above 2400 N for some configurations during the prescribed training schedule.

TransformerConv and GATConv incorporate elastic interaction coefficients directly into their attention operations. The unmodified GINConv implementation instead usessum aggregation without direct edge weighting in this implementation. GINConv obtained a transfer RMSE of 232.7 N at L=2, whereas its RMSE exceeded 2900 N at L=3 under the tested settings, as shown in Figure 7.

TransformerConv-L3 was selected by jointly examining transfer RMSE, UQ NLL and interval coverage on the augmented-sample subset used in Figure 7 and Figure 8. It obtained an RMSE of 170.9 N, an NLL of 8.839 and nominal 95% coverage of 93.9%. TransformerConv-L4 achieved a lower RMSE of 140.2 N, together with an NLL of 8.873 and coverage of 90.0%. Because this subset also supported architecture selection and contains related sequence variants, the values are reported as development metrics. A source-grouped validation/test design will provide the corresponding independent performance estimate.

Mechanically, each previously tightened bolt can receive load redistribution messages from several bolts tightened later in the sequence. TransformerConv assigns condition-dependent attention to these directed interactions while incorporating the sign and magnitude of the EICM edge attribute. This representation matches the many-to-one causal structure of sequential tightening more directly than one-dimensional recurrent encoding, while residual connections preserve each bolt’s local state across message-passing layers. The observed development-split advantage is consistent with this mechanical interpretation.

Performance varied with network depth, as shown in Figure 8. GATConv obtained its lowest transfer RMSE at three layers (275.3 N), whereas its lowest NLL and closest coverage to the nominal target occurred at two layers (92.6%). SAGEConv improved from 347.2 N at two layers to 235.6 N at three layers and 234.3 N at four layers. LinearConv yielded transfer RMSE values of 236.0 N and 187.1 N at two and four layers, respectively, but did not converge during elastic pre-training for some deeper configurations. The tested aggregation mechanisms, therefore, required architecture-specific depth selection.

### 4.3. Ablation Study on the Transfer Learning Strategy

We conducted an ablation study to examine the contribution of each stage in the progressive training strategy. Four training configurations were defined by selectively bypassing individual stages. Their operational definitions are summarized in Table 4.

**Figure 10:**
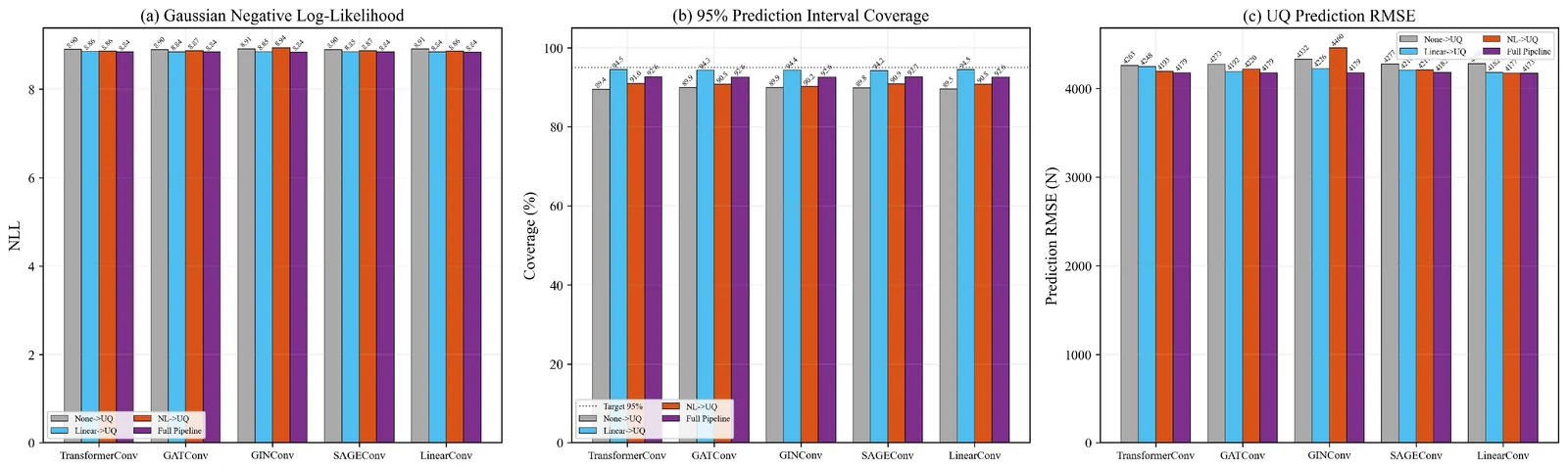
Single-run ablation metrics for the staged training configurations. Exact bar values are annotated; percentage RMSE changes are reported in the accompanying text.

Figure 10 compares the four training configurations across the five evaluated architectures.

Bypassing both deterministic pre-training stages increased prediction RMSE for every tested architecture. The UQ_only configuration trains directly on the UQ dataset without physics-informed initialization; for GINConv, its prediction RMSE reached 4332 N and its NLL was 8.912. These results show that the two deterministic stages provide essential initialization for learning from the available UQ benchmark.

For TransformerConv, the Linear + UQ and NL + UQ configurations produced similar prediction RMSE values of 4193 N and 4190 N, respectively. Their interval coverage differed: Linear + UQ yielded 94.3%, whereas NL + UQ yielded 90.8%. This comparison suggests that elastic pre-training affected the fitted interval widths in the evaluated split, but broader calibration claims require independent data.

The full pipeline provided the lowest combined errors among the tested ablations. The results are consistent with the intended role of elastic pre-training as an initialization for spatial coupling and nonlinear fine-tuning as a correction for the sealed-flange response. Because the stages use related augmented samples from a single flange configuration, the ablation establishes a within-dataset contribution rather than independent generalization.

### 4.4. Effectiveness of Physical Prior Knowledge as Edge Features

We assessed the contribution of the elastic interaction coefficients by comparing weighted models with a binary-graph baseline, in which every existing edge had a weight of 1.0. Four representative configurations were tested: TransformerConv at L=4, GATConv at L=3, SAGEConv at L=4, and GINConv at L=2. Hyperparameters and random seeds were held constant within each comparison.

**Figure 11:**
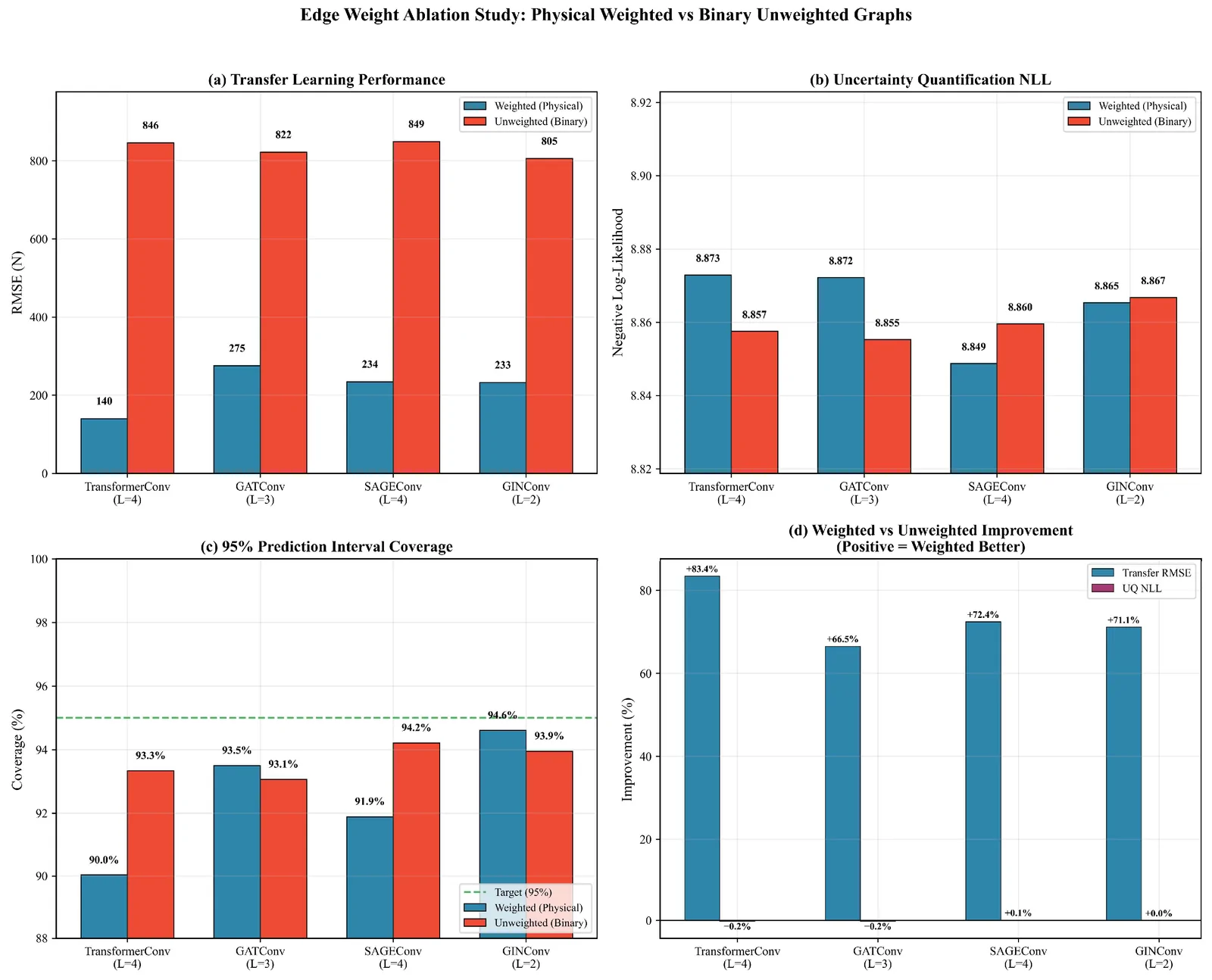
Single-run comparison of signed EICM edge attributes and binary edge attributes. Exact values and percentage RMSE reductions are reported in the text, and the caption identifies the evaluation basis for direct interpretation. In panel (**d**), the purple series represents the change in UQ NLL.

As shown in Figure 11, physical edge weights reduced transfer RMSE for all four tested configurations. TransformerConv decreased from 845.9 N to 140.2 N, a reduction of 83.4%. The reductions were 72.4% for SAGEConv (849.0 to 234.3 N), 71.1% for GINConv (805.4 to 232.7 N), and 66.5% for GATConv (821.8 to 275.3 N). The mean reduction was 73.4% across these configurations.

The binary graph treats all connected bolt interactions equally, whereas the elastic coefficients preserve their magnitude and sign. This difference provides a plausible explanation for the observed error reductions. TransformerConv showed the largest reduction, consistent with its direct integration of edge features into the attention mechanism.

The edge representation also changed the UQ metrics. For example, weighted GINConv yielded 94.6% coverage for the nominal 95% interval and an NLL of 8.865. These values characterize calibration behaviour on the reported development split and further demonstrate the influence of signed mechanics-informed attributes.

### 4.5. Cross-Method Comparison with Sequential Baselines

We compared the selected three-layer TransformerConv model with two LSTM variants and two Elman RNN variants. All methods used the same three-stage multi-fidelity pipeline. The Enhanced recurrent variants received the elastic interaction coefficient between consecutive bolts as an additional input, whereas the Base variants used only preload and normalized tightening order. The comparison, therefore, evaluates both representation type and the inclusion of a physics-informed feature within the tested data split.

Table 5 reports the same-split numerical comparison of the proposed GNN, recurrent baselines, and the EICM analytical prior.

Table 6 provides a contextual comparison with representative published studies. Its values answer different scientific questions and retain their original datasets, units, and task definitions; the comparison is therefore qualitative rather than directly numerical.

The timing environment was as follows: PyTorch 2.8.0, NVIDIA GeForce RTX 5070, random model weights and a full causal directed graph. Fixed launch overhead dominates the GPU measurements at these graph sizes. A future paired benchmark will compare these inference times with completed FE cases under matched geometry, loading, solver settings and hardware.

**Figure 12:**
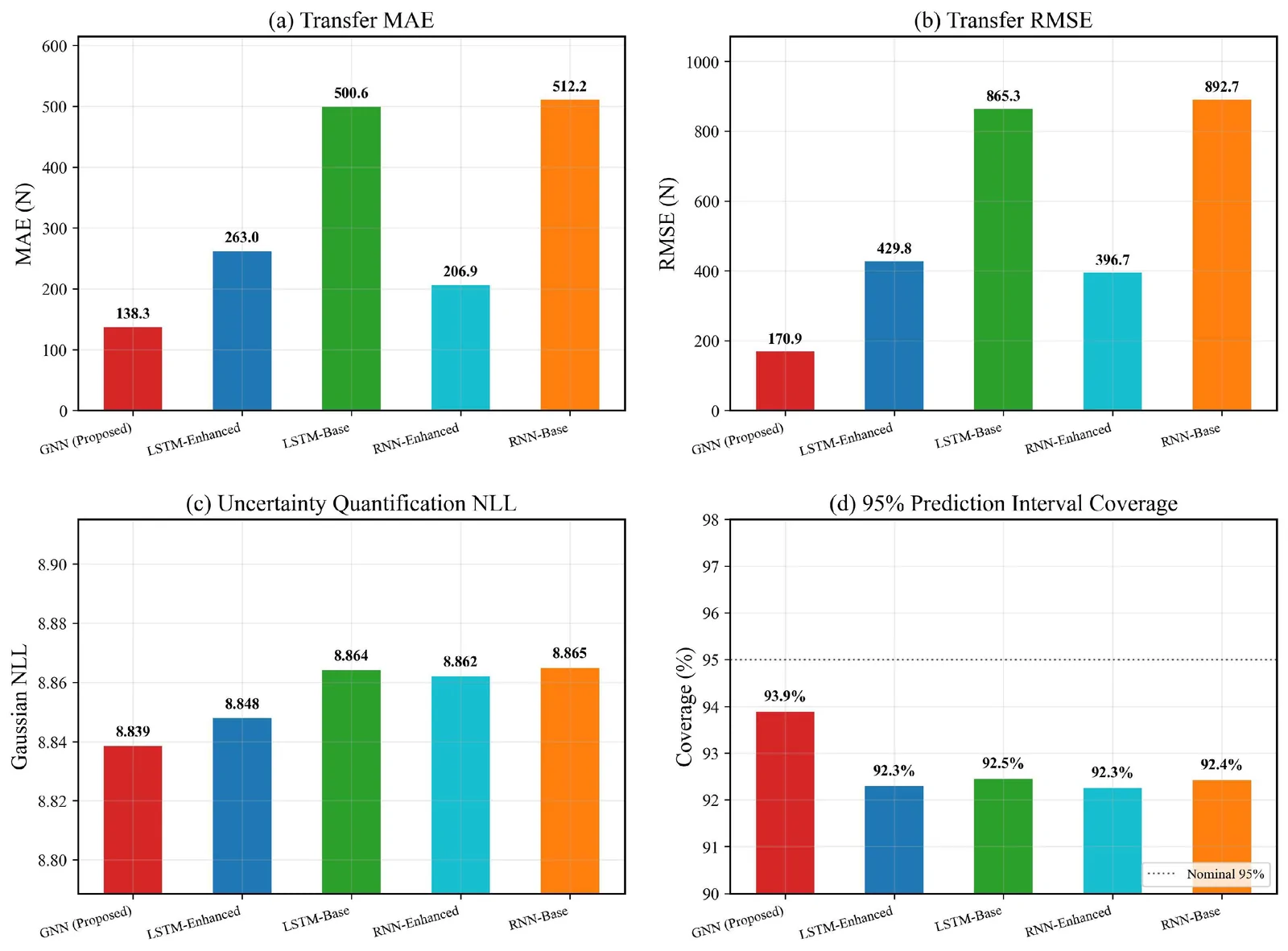
Development-split comparison of TransformerConv-L3, two LSTM variants, and two RNN variants for MAE, RMSE, NLL, and nominal 95% prediction-interval coverage. Table 6 provides the corresponding task-level context for representative published studies.

**Figure 13:**
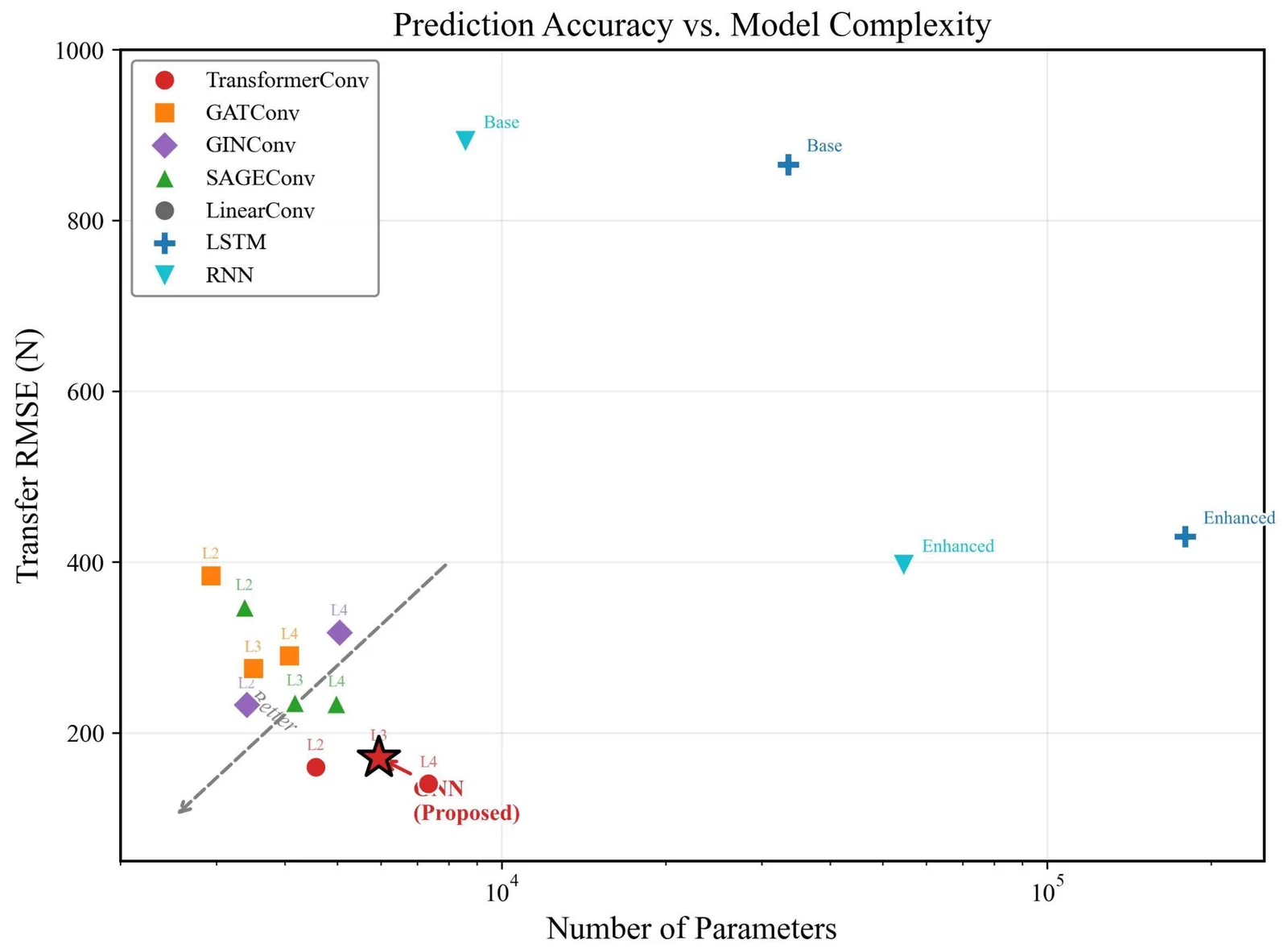
Development-split RMSE versus parameter count for the evaluated architecture-depth combinations. The star identifies TransformerConv-L3 as the selected balance between predictive accuracy, interval behaviour, and model size.

**Figure 14:**
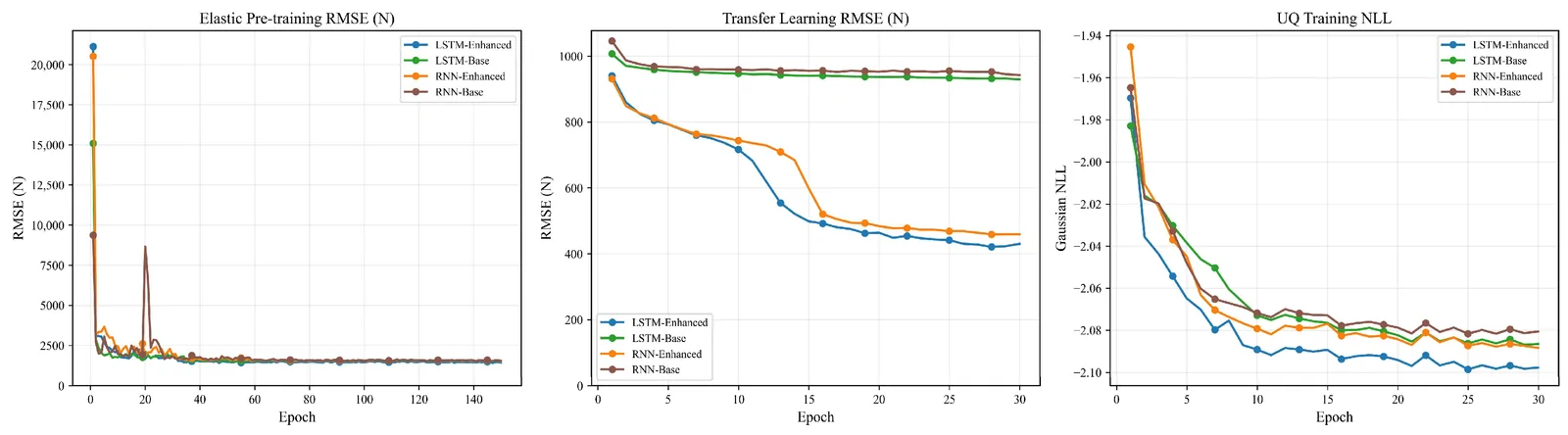
Training curves for the LSTM and RNN baselines across EICM pre-training, nonlinear-discrepancy transfer, and UQ-head fitting with ultrasonic measurements. Table 6 supplies the broader literature context using each study’s original task and metric.

Table 5 and Figure 12 summarize the quantitative comparison. Figure 13 relates transfer RMSE to parameter count, and Figure 14 shows the training curves of the recurrent models.

Within the augmented development split, TransformerConv-L3 obtained an RMSE of 170.9 N, compared with 396.7 N for RNN-Enhanced and 679.3 N for the EICM analytical prior. The GNN error corresponds to 0.31% of the 55 kN normalization preload and 0.28–0.34% of the 50–60 kN input range. This percentage provides a clear numerical scale for the benchmark. Engineering acceptance will additionally require application-specific preload tolerances and a validated relationship among bolt load, contact pressure and leakage performance.

Adding elastic interaction coefficients reduced transfer RMSE from 865.3 N to 429.8 N for LSTM and from 892.7 N to 396.7 N for RNN. The corresponding reductions were 50.3% and 55.6%. Thus, the physics-informed feature was beneficial for both recurrent baselines tested.

RNN-Enhanced obtained a lower transfer RMSE than LSTM-Enhanced (396.7 versus 429.8 N). Under the reported 60-bolt sequence representation and hyperparameters, recurrent gating offered no additional accuracy. A broader tuning study will further separate architecture and optimization effects.

The selected GNN used 5953 parameters, approximately 30-times fewer than LSTM-Enhanced and 9.2-times fewer than RNN-Enhanced. In Figure 13, this configuration combines the lowest transfer RMSE with the smallest parameter count among the enhanced models. The compact model size is consistent with explicitly encoding bolt topology rather than learning it solely from a sequence vector.

Table 5 reports end-to-end training times for the three computational stages. Table 7 separately evaluates forward-pass cost as graph size increases. The three TransformerConv layers, feature dimensions, and 5953 model parameters are held fixed, while the node count increases from 60 to 120 and 240. The corresponding causal edge counts are 1770, 7140, and 28,680. These measurements quantify architecture-level inference scaling and do not assess predictive accuracy for the larger node counts.

Figure 14 shows that the recurrent models ended elastic pre-training with RMSE values of 1431–1537 N, compared with 133 N for the GNN. During nonlinear fine-tuning, the recurrent variants plateaued at higher errors within the prescribed 30 epochs. Enhanced variants had lower losses than their Base counterparts in both recurrent families under the tested training settings.

### 4.6. Uncertainty Quantification Reliability Analysis

We examined the prediction intervals on the UQ development split using a reliability diagram and a representative 60-bolt measurement condition. These diagnostics evaluate interval consistency against repeated ultrasonic measurements.

**Figure 15:**
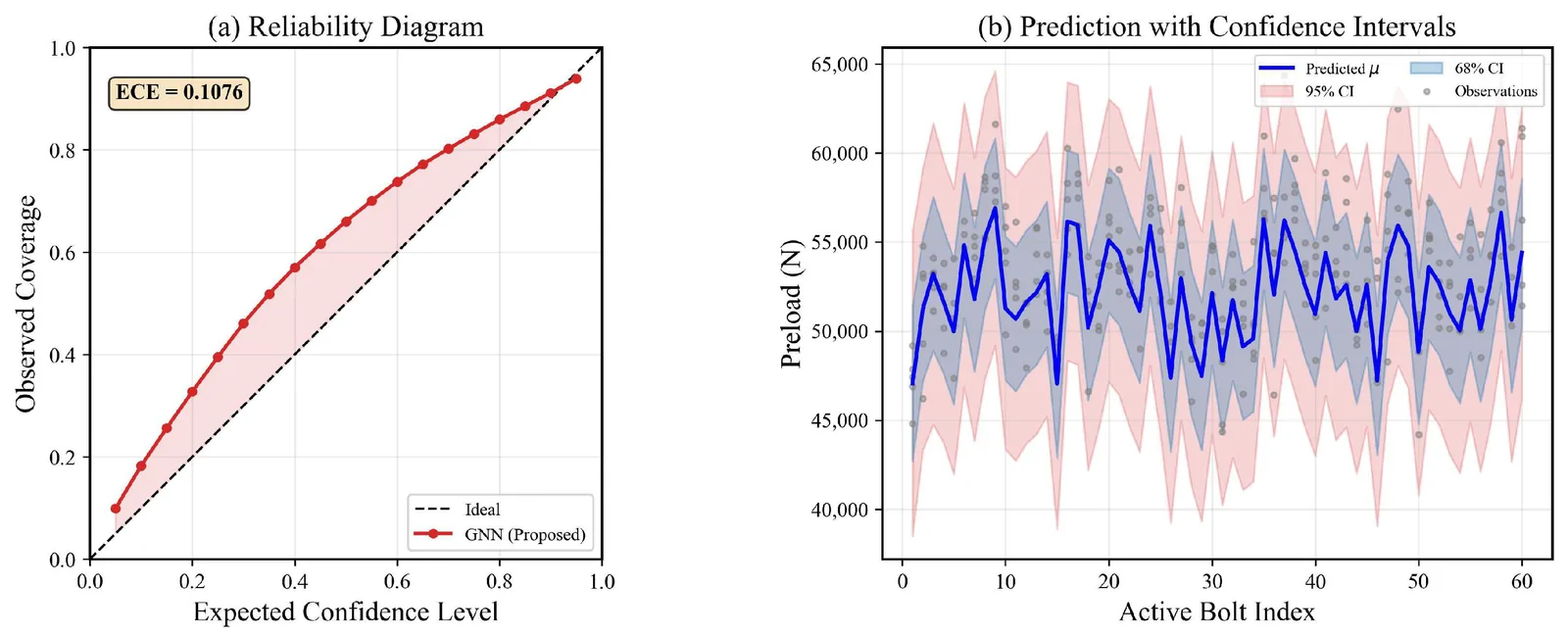
Uncertainty diagnostics for TransformerConv-L3 on the UQ development split: (**a**) observed versus nominal prediction-interval coverage with mean absolute coverage error; (**b**) predicted mean with 68% and 95% prediction intervals for all 60 bolts in one representative condition, together with five ultrasonic measurement replicates per bolt.

Figure 15a evaluates 19 nominal central prediction intervals from 5% to 95% in 5-percentage-point increments. The mean absolute difference between nominal and observed coverage was 0.1076.

Coverage of the nominal 95% interval was 93.9% on the 162-instance UQ development subset. Across the 19 evaluated interval levels, the mean absolute coverage deviation was 0.1076.

Figure 15b displays all 60 bolts from one representative measurement condition, with five ultrasonic measurement replicates per bolt and the model’s 68% and 95% prediction intervals. The changing interval widths reflect input-dependent measurement variability.

## 5. Conclusions

This study presented a physics-informed graph-learning framework for sequence-aware residual preload prediction in a 60-bolt flange. The full causal directed graph retains every later-to-earlier tightening dependency, while local signed EICM coefficients provide an interpretable mechanics prior for 240 of the 1770 edges.

The workflow integrates EICM pre-training, nonlinear-discrepancy transfer, and heteroscedastic modelling of ultrasonic preload measurements, providing a coherent route from low-cost mechanics to residual-field prediction and interval estimation.

On the augmented development split used for model selection, TransformerConv-L3 achieved an RMSE of 170.9 N, an NLL of 8.839, and 93.9% coverage of the nominal 95% prediction interval. Across four internal architecture configurations, incorporating physical edge features reduced RMSE by an average of 73.4%. Compared with the lowest-error recurrent baseline evaluated on the same split, the graph model reduced RMSE by 56.9% while using only 5953 parameters. Together, these results demonstrate the effectiveness of explicit causal topology and signed interaction attributes within the evaluated 60-bolt benchmark.

### Limitations and Future Work

First, the reported metrics characterize the nominal 60-bolt benchmark. A broader test matrix should combine source-grouped and leave-one-strategy-out evaluation with labelled cases covering different flange sizes, bolt numbers, materials, seals, preload levels, FE parameters, and service conditions. The fixed architecture executes the 120- and 240-node graphs reported in Table 7, but these timing results do not establish predictive generalization. Accuracy across bolt counts requires labelled cross-configuration cases within the broader test matrix.

Second, additional assembly groups with strain-, load-cell-, or load-washer-based references would enable independent assessment of ultrasonic measurement repeatability and preload accuracy.

Finally, the uncertainty framework can be extended to epistemic uncertainty, FE-model discrepancy, and distribution shift through deep ensembles, perturbed or missing inputs, and validation-fitted interval calibration.

## Figures and Tables

**Table 1:** Nominal Mooney–Rivlin parameters for the motivating seal specification.

Component	$C_{10}$ (MPa)	$C_{01}$ (MPa)	$D_{1}$ (${MPa}^{-1}$)
Sealing Ring 1	0.5675	0.2836	0
Sealing Ring 2	1.8700	0.4700	0

**Table 2:** Extracted elastic interaction coefficients for edge feature initialization.

Elastic Interaction Coefficient	$A_{1,2}$	$A_{1,3}$	$A_{1,4}$	$A_{1,5}$	$A_{1,57}$	$A_{1,58}$	$A_{1,59}$	$A_{1,60}$
value	−0.0499	0.0039	0.0007	0.0001	0.0003	0.0012	0.0007	−0.0499

**Table 3:** Hyperparameter settings for the progressive three-stage training pipeline.

Parameter	Phase 1 (Elastic)	Phase 2 (Nonlinear)	Phase 3 (UQ)
Learning Rate	0.01	$10^{-4}$	$10^{-3}$
Epochs	150	30	30
Training Samples	Online (50/epoch)	1458	1458
Optimizer	Adam	Adam	Adam
Loss Function	MSE	MSE	NLL
Trainable Parameters	Entire GNN	Entire GNN	UQ Head Only

**Table 4:** Operational definitions of the ablation study configurations.

Configuration	Phase 1: Elastic Pre-Training	Phase 2: Nonlinear Transfer	Phase 3: UQ Fine-Tuning
Full Pipeline			
Linear + UQ		×	
NL + UQ	×		
UQ_only	×	×	

**Note:** × indicates that the corresponding training phase was omitted (bypassed) in that ablation configuration.

**Table 5:** Cross-method comparison of GNN, LSTM, and RNN for bolt preload prediction.

Method	Parameters	Transfer MAE (N)	Transfer RMSE (N)	UQ NLL	95% Coverage (%)	Time (s)
**GNN (Proposed)**	5953	**138.3**	**170.9**	**8.839**	**93.9**	1397
LSTM-Enhanced	179,009	263.0	429.8	8.848	92.3	855
LSTM-Base	33,537	500.6	865.3	8.864	92.5	760
RNN-Enhanced	54,593	206.9	396.7	8.862	92.3	822
RNN-Base	8577	512.2	892.7	8.865	92.4	746
EICM analytical prior	—	635.7	679.3	n/a	n/a	analytical

**Note:** — indicates not applicable (no trainable model parameters); n/a indicates that the metric is unavailable because the analytical EICM prior does not produce probabilistic outputs. Bold type identifies the proposed method and the best metric values among the compared learning-based models.

**Table 6:** Contextual comparison with representative published studies. Metrics are retained in their original task-specific form to support qualitative positioning across studies.

Reference	Task and Data	Method	Published/Reported Metric	Direct Comparability
Xu et al. [10]	Single-bolt preload from hydraulic pressure and nut angle	Two-stream deep network	Preload deviation reduced to approximately 10%	No: different inputs, target and validation data
Li et al. [40]	Bearing fault classification on NEFU and HUST datasets	MCFormer multi-sensor Transformer	99.50% and 98.33% classification accuracy	No: classification rather than residual preload regression
Present work	Sequence-conditioned 60-bolt residual preload benchmark with ultrasonic measurements	TransformerConv-L3	MAE 138.3 N; RMSE 170.9 N; nominal-95% coverage 93.9%	Development-split benchmark

**Table 7:** Forward-pass timing as graph size increases for a fixed three-layer TransformerConv architecture. The feature dimensions, layer count, and 5953 parameters remain unchanged; only the node and edge counts increase. Each entry is the median of 200 repeated calls. The 120- and 240-node cases evaluate computational graph-size scaling, not predictive accuracy for larger flanges.

Graph Nodes, N	Causal Edges	Non-Zero Prior Edges	CPU Median (ms)	GPU Median (ms)
60	1770	240	5.416	2.247
120	7140	480	7.893	2.196
240	28,680	960	12.647	1.883

## Data Availability

Data will be made available upon request.
